# Psychosocial predictors of antenatal stress in Pakistan: perspectives from a developing country

**DOI:** 10.1186/s13104-020-05007-3

**Published:** 2020-03-18

**Authors:** Ahmed Waqas, Muhammad Zubair, Sadiq Zia, Hafsa Meraj, Kapil Kiran Aedma, Muhammad Hassan Majeed, Sadiq Naveed

**Affiliations:** 1 CMH Lahore Medical College and Institute of Dentistry, Lahore Cantt, Pakistan; 2 FMH College of Medicine and Dentistry, Lahore, Pakistan; 3 Zia Hospital, Sadiqabad, Pakistan; 4 Shifa College of Medicine, Islamabad, Pakistan; 5 Unity Point Health Methodist, Peoria, IL 61636 USA; 6 Quinnipiac University Hamden, Norwich, CT USA; 7 Kansas University Medical Center, Kansas City, USA

**Keywords:** Gender discrimination, Stress, Pregnancy, Obstetric treatment, Health inequalities

## Abstract

**Objective:**

Antenatal stress is highly prevalent globally and is associated with adverse physical and psychiatric morbidities and adverse neonatal outcomes. However, the burden of antenatal stress and its psychosocial predicators have not been explored in context of the Pakistani sociocultural environment. The present study explores the prevalence of antenatal stress and its association with gender of offspring, socioeconomic background, cultural beliefs, and access to healthcare in the province of Punjab, Pakistan.

**Results:**

There was a total of 516 pregnant women. Antenatal stress was measured by the Perceived Stress Scale (PSS). The mean score of the respondents on the Perceived Stress Scale was 7.55 (3.43). A total of 218 (42.2%) respondents reported higher stress levels. Logistic regression analysis (backward method) yielded a significant model predicting high stress levels. According to it, low family income, unplanned pregnancy, increasing number of children, less autonomy in decision making, marital problems, harassment, desire to have a male offspring, and the history of birth complications, attended by midwives were associated with high stress levels.

## Introduction

For most women, pregnancy is an enjoyable and pleasant experience, but for many it can open a window to chronic stress due to pregnancy-related biopsychosocial and cultural stressors. Antenatal stress is a major concern in low and middle-income countries (LMIC), with a prevalence of 29–60%, owing to a higher risk of social, psychological, and physical hardships [1–4]. It is associated with many psychopathological issues among women including perinatal anxiety and depression, suicidality, eating disorders, and increased fear of childbirth [5–7]. It is also associated with many birth and developmental abnormalities among the offspring such as growth retardation, emotional and behavioral problems, neurodevelopmental disorders, preterm births and poor APGAR scores [8–11].

Pakistan is the sixth most populous country globally, with a population exceeding 200 million. It boasts a unique cultural identity in Asia [12]. Pakistan has its own distinct culture, languages, food, gender disparities, and, most importantly, cultural traditions revolving around pregnancy that moderate antenatal care. Unfortunately, Pakistan is also struggling with several demographic and socio-economic stressors, frequent infectious outbreaks, terrorism, and traumatic events such as wars and internally displaced refugees that are among common reasons for the high burden of psychopathologies [13, 14]. Hence, it presents a very peculiar set of biopsychosocial factors that can precipitate high levels of perinatal stress [3].

Due to the detrimental impact of antenatal stress, it is important to explore the prevalence and factors related to it. Factors related to treatment seeking attitudes, access to healthcare, and preference of birth-related procedures have been shown to correlate with common mental health disorders in Pakistan as well as in other countries [4, 12]. Most of the world’s population lives in LMIC, boasting the highest population growth rates. Unfortunately, there are only a few studies focused on exploring antenatal stress in the sociocultural context of the developing world. Therefore, this study was designed to explore the prevalence of prenatal stress and its association with socioeconomic background, cultural beliefs, disparities related to gender of offspring such as desire for a son and access to healthcare in the province of Punjab, Pakistan.

## Main text

### Methods

From November 2016 to March 2017, a cross-sectional study was conducted at two locations: the District of Faisalabad and Charanwala Village in the District Mandi Bahauddin. Faisalabad is a major metropolitan city in Punjab, Pakistan. Study participants were recruited from two teaching hospitals in Faisalabad, namely: District headquarter hospital and Children Hospital. The third site was the Basic Health Unit in Charanwala village in the district Mandi Bahauddin. The former two sites are the only secondary and tertiary care and publicly funded hospitals, providing obstetric and gynecological care to urban and rural population residing within Faisalabad and adjoining rural areas. While the Basic Health Unit in Charanwala village caters to the primary health care needs of the rural families. These three sites thus, provided a homogeneous study sample, representative of both the urban and rural population in the province of Punjab.

A systematic sampling procedure was used, where every fifth patient presenting at obstetrics and gynecology departments of the selected hospitals were interviewed by a six-member team consisting of physicians, nurses and medical students. Ethical approval was sought and received from FMH College of Medicine and Dentistry, Lahore, Pakistan (FMH-06-2017-IRB-261-M). Participation in this study was voluntary and all participants signed the informed consent form.

All respondents were interviewed using the preformed questionnaire consisting of four parts: (1) variables related to demographic characteristics and obstetric history; (2) a 4 item Perceived Stress Scale (PSS-4) and (3) an Urdu version of Pittsburgh Sleep Quality Index (PSQI-U). Results for sleep pattern based on PSQI scale have been reported in another study (Waqas et al. In press).

The section on demographic characteristics enquired information pertaining to age, background, religion, ethnicity, socioeconomic class and education. The second section of the questionnaire pertained to obstetric variables including parity, status of pregnancy, trimester, previous complications during past deliveries by hospital staff and untrained midwives, any comorbidities, and gender of previous offspring and gender of fetus as evident on ultrasonography. While the third section included variables related to family dynamics included household decision maker, preference for education for daughters and sons, gender preference for fetus among in-laws, experience of marital problems, harassment and domestic abuse. These items were asked using close ended question format.

Perceived Stress Scale (PSS-4) scores are obtained by reverse coding the positive items, and then summing across all 4 items. It exhibited a good internal consistency in the present study sample (Cronbach’s alpha = .793). As indicated in previous studies, stress levels were dichotomized into low and high, by combining upper and lower two quartiles [15, 16].

#### Statistical considerations

Total sample size for this study was estimated to explore several research questions. To estimate prevalence of high stress levels during pregnancy, a minimum sample size of 385 was required based on following parameters: 95% confidence level, 5% margin of error and a population of 33,85,000 in Faisalabad. For logistic regression analyses, most conservative estimates recommend 20 participants per predictor as a rule of thumb, thus, indicating a sample size of 240 to 300 as appropriate for a model comprising of 12–14 predictors. All data were analyzed in SPSS v.20 (IBM, Chicago, IL). Categorical variables were presented as frequencies and continuous data as mean (SD). Logistic regression analysis (backward method) was run with covariates demonstrating significant univariate associations, to identify the significant predictors of antenatal stress in our study sample. Introduction of predictors in the regression model was guided by significance levels of the bivariate analysis between PSS-scores and different variables, biological plausibility and previous literature (4).

### Results

Out of 550 respondents, 516 consented to participate in the study yielding a response rate of 93.82%. The mean age of respondents was 29.82 (6.50) years. Among socioeconomic background, most of the respondents were middle class 364 (70.54%), followed by low 135 (26.2%), and upper class 17 (3.3%). Most of the respondents were educated from 5th to 10th grade 260 (50.39%), followed by illiterate 134 (26%), and undergraduates 122 (23.64%). A total of 260 respondents (50.4%) belonged to rural areas, followed by semi-urban (185, 35.9%) and urban areas (71, 13.8%). Most were house-wives (426, 82.6%), while the rest were either hobbyists or employed. A majority were natives of Punjab (395, 76.6%) while the rest had migrated from other provinces.

The mean score of the respondents on Perceived Stress Scale was 7.55 (3.43). A total of 218 (42.2%) respondents reported higher stress levels. The mean number of offspring borne by women were 2.32 (2.1). Many respondents reported unplanned pregnancies, a history of miscarriage (n = 101, 19.6%) and self-induced termination of pregnancy (n = 50, 9.7%). A higher proportion of women had past delivery procedures performed at hospitals (n = 317, 61.43%). However, the respondents reported a significant percentage of past delivery procedures performed by midwives (n = 256, 44.6%) as well. A higher proportion of respondents reported complications during birth procedures performed by midwives (51.95%) than physicians (17.98%). A significant percentage of respondents reported having chronic illnesses (Table 1).

**Table 1:** Response distribution to variables related to obstetric history

Variable	Subcategory	Frequency	Percentage (%)	Mean (SD)	χ^2^ statistic	P-value
Trimester	1st	123	23.8		51.78	< .001
	2nd	248	48.1			
	3rd	145	28.1			
Pregnancy	Planned	303	58.7		15.70	< .001
	Unplanned	213	41.3			
Complication during past deliveries in hospital	Yes	57	11.1		313.19	< .001
	No	459	88.9			
Complication during past deliveries by midwives	Yes	133	25.8		121.12	< .001
	No	383	74.2			
Any chronic illness	Yes	111	21.5		167.51	< .001
	No	405	78.5			
Number of live births				2.32 (2.1)		
Number of still births				.295 (.84)		
Terminated pregnancies				.122 (.41)		
Number of sons				1.06 (1.21)		
Number of daughters				1.24 (1.38)		
Age of the eldest child				6.24 (5.51)		
Children delivered in hospitals				1.31 (1.37)		
Children delivered by midwives				1.38 (2.09)		

Most of the respondents reported that their husbands were the primary household decision makers followed by their in-laws and themselves. Most of the respondents reported no preference for the gender of their child, however, a high proportion preferred son over daughters by themselves and their in-laws. Most of the respondents did not report any preference of education based on gender of offspring. A high percentage of respondents reported having relationship problems with their husbands, experience of harassment as well as domestic abuse (Table 2).

**Table 2:** Response frequency to variables highlighting gender related disparities and preference for sons

	Frequency (n)	Percentage (%)	χ^2^ statistic	P-value
Household decision maker				
Self	46	8.9	228.50	< .001
Husband	323	62.6		
In-laws	147	28.5		
Ultrasound				
Yes	153	29.7		
No	363	70.3		
Gender on ultrasound				
Male	84	55.6		
Female	67	44.4		
Gender preference				
Male	177	34.3	213.71	< .001
Female	34	6.6		
No preference	305	59.1		
Preference for education				
Sons	47	9.1	717.11	< .001
Daughters	11	2.1		
No preference	458	88.8		
In-laws prefer sons				
Yes	194	37.6	31.75	< .001
No	322	62.4		
Marital problems				
Yes	127	24.6	133.03	< .001
No	389	75.4		
Harassment				
Yes	139	26.9	109.78	< .001
No	377	73.1		
Domestic abuse				
Yes	96	18.6	203.44	< .001
No	420	81.4		

Experience of harassment had significant association with an increasing number of daughters (r_pb_ = .231, P < .001) self-induced abortions (r_pb_ = .237, P < .001) and miscarriages (r_pb_ = .151, P < .001). Experience of domestic abuse also exhibited significant association with an increasing number of daughters (r_pb_ = .232, P < .001) self-induced abortions (r_pb_ = .163, P < .001) and miscarriages (r_pb_ = .182, P < .001).

The logistic regression model (backward method) predicted 71.5% of the model correctly. A non-significant Hosmer and Lemeshow test represented a good model fit, explaining 29% of variance in PSS-scores. According to it, high stress levels among pregnant women were associated with low-income status, non-involvement of the respondent in household decision making, desire for a son than a daughter or having no preference, experience of birth complications in past pregnancies by midwives, and experience of marital problems with husband and previous experiences of harassment (Table 3).

**Table 3:** Gender disparity and obstetric history as predictors of high stress levels among pregnant women (n = 516)

Predictors	Subcategories	B	Std. error in B	P-value	Odds ratio	95% C.I. for OR	
						Lower	Upper
Household income		.353	.142	.013	1.423	1.077	1.879
Household decision maker	In-laws			.050			
	Husband	− .952	.434	.028	.386	.165	.904
	Self	− .444	.241	.066	.641	.400	1.029
Pregnancy	Unplanned/planned	− .797	.246	.001	.450	.278	.730
Number of children		− .129	.061	.033	.879	.780	.989
Gender preference	No preference			.011			
	Male	.319	.233	.172	1.375	.870	2.173
	Female	− 1.398	.583	.016	.247	.079	.775
Complications during birth procedure by mid-wives	Yes/no	.466	.273	.088	1.594	.933	2.722
Marital problems	Yes/no	.779	.338	.021	2.178	1.124	4.223
Harassment	No/yes	1.188	.322	.000	3.280	1.745	6.164
Constant		− .621	.437	.155	.538		

Hosmer and Lemeshow test statistic = 3.54, P = .896, Cox and Snell R^2^ = .22, NegelKerke R^2^ = .29, Model Chi^2^ = 113.50, P < .001

### Discussion

The present study reports a very high prevalence of antenatal stress in Pakistan, which is substantiated by previous studies conducted in the region. A prevalence ranging from 29% to as high as 66% has been identified within Pakistan and neighboring countries, highlighting the high burden of antenatal stress in LMIC, moderated by region specific sociocultural stressors [1–4].

Our study identifies potential household dynamics and sociocultural stressors that moderate levels of antenatal stress in Pakistan, including low household income, marital problems and experience of harassment. Our findings are corroborated by a number of previous studies conducted in the South Asian region [17, 18]. The lack of autonomy in household decision-making and healthcare was found to be a strong predictor of antenatal stress. Autonomy in health care is considered the right to make informed medical decisions about oneself. It becomes imperative during a pregnancy when the mother has to make decisions about herself and the fetus [19]. Moreover, the lack of autonomy in running everyday household affairs leads to a dependency on in-laws. Restrictions on autonomy, cultural constraints, and limitations in making household decisions are a pervasive problem in Pakistan, and hence a potential cause of antenatal stress [20].

In our study sample, most women had undergone birth procedures in a hospital setting. However, almost fifty percent of our respondents had undergone birthing procedures by midwives, associated with a higher proportion of complications and antenatal stress than at hospitals. Most of these complications occurred at home because of birth procedures performed by low trained midwives called “Dai” in Urdu & Punjabi language. This finding further strengthens the evidence in the literature that an adverse outcome during previous pregnancy is a risk factor for prenatal stress [12]. These traumatic memories can haunt a mother and can precipitate stress in subsequent pregnancies. Moreover, respondents undergoing abortions and miscarriages also reported episodes of harassment and domestic abuse, highlighting the cultural stigma revolving around genetic defects, abortions and poor health information seeking attitudes [21]. This adds insult to the injury and further potentiates the psychological effects of these traumatic events [4].

Our results reveal that the number of pregnancies is a distinct indicator for antenatal stress. The exact reason for these findings is unknown but it could be related to less excitement and novelty after many earlier pregnancies. Moreover, Islam, the dominant religion in the country, along with sociocultural beliefs discourage termination of pregnancies and contraception, translating to high birth rates in the region [4]. With increasing number of children mothers may be anxious about problems related to raising more children, poor maternal and child health outcomes and finances associated with childcare [22]. Grand multigravidity (more than 5 children) is also a known independent predictor of abortion, causing excessive Antenatal stress among women [22]. In addition, we found that unplanned pregnancies predicted high levels of stress among women. In general, poor knowledge and practice of methods of contraception among women and less autonomy to make decisions about family planning can lead to more unplanned pregnancies [22]. An unintended pregnancy can cause an excessive psychological burden on expectant mothers as it demands access to better health care, improved nutrition, physical rest and may cause financial stress on the family [23, 24].

Our study also explored gender disparities and stigmas that revolve around pregnancy in our region. According to our analysis, preference for male children was associated with high stress levels in our study sample. This gender preference behavior has also been highlighted in previous studies conducted in Lahore and Karachi, Pakistan [4, 25, 26]. While the expectation of in-laws for a male child is culturally accepted by a mother, it can put undue stress on her. In rural Pakistan, contrary to all scientific knowledge, it is a commonly held belief that a woman’s egg, not the father’s semen, determines the gender of the fetus [1]. This notion sometimes leads to men practicing polygamy in the society. However, in our study, mother’s equal interest in a child’s education irrespective of the gender is a welcoming finding, in a country that is trying to close the gender parity gap in education. But discrimination still remains with women giving birth to more daughters, reporting harassment and domestic abuse at their homes [26, 27].

## Limitations

Self-reporting nature of the questionnaires may introduce recall bias in the study. Moreover, due to sensitive nature of the information, respondents might have underreported information about domestic violence. Due to its cross-sectional design, causal relationships cannot be established between two variables. Despite a higher sample size and an inclusive study sample, the results of this investigation should not be generalized to the whole Pakistani population. In addition, experiences of domestic violence and harassment by expectant mothers were inquired using dichotomous questions, which may not be a sensitive measure of these constructs.

## Data Availability

A confidentiality agreement with participants prevents us from sharing the data, therefore, dataset cannot be shared.

## Abbreviations

LMIC: Low and middle income countries
PSQI-U: Urdu version of Pittsburgh Sleep Quality Index
PSS-4: Perceived Stress Scale
